# How a Preschool Intervention Affected High School Outcomes: Longitudinal Pathways in a Randomized‐Controlled Trial

**DOI:** 10.1111/cdev.14235

**Published:** 2025-04-01

**Authors:** Karen L. Bierman, Brenda S. Heinrichs, Janet A. Welsh, Damon E. Jones, D. Max Crowley

**Affiliations:** ^1^ The Pennsylvania State University University Park Pennsylvania USA

**Keywords:** adolescent outcomes, early intervention, follow‐up studies, randomized‐controlled trial, social–emotional learning

## Abstract

This study examined the impact of the Head Start Research‐based, Developmentally Informed (REDI) preschool intervention on high school outcomes and explored longitudinal mediation. 356 children (58% White, 25% Black, 17% Latinx; 54% female, 46% male; *M*_age_ = 4.49 years) were recruited from Head Start classrooms which were randomized to intervention (*N* = 192) or “usual practice” (*N* = 164). REDI effects emerged on high school emotional symptoms (teacher ratings, *d* = 0.41) and behavior problems (composite of teacher, parent, youth ratings, *d* = 0.27) with the latter benefits mediated by earlier intervention boosts to social–emotional learning, social adjustment, and parent involvement. REDI had no direct impact on GPA or on‐time graduation but promoted these outcomes indirectly mediated by earlier intervention effects.

## 1 Introduction

Head Start was initiated in 1965 to address socioeconomic disparities that affect early development and reduce children's educational attainment and overall well‐being (Office of Head Start 2021). Since that time, public investment in early childhood education (ECE) has expanded dramatically in the United States; 45 (of 50) states now fund preschool programming (Friedman‐Krauss et al. 2023). The expansion of ECE was motivated in part by evidence documenting long‐term benefits (reduced problem behaviors, improved mental health and educational attainment) for children from low‐income families attending high‐quality ECE programs (Perry Preschool; Abecedarian Project) relative to at‐home care (Friedman‐Krauss et al. 2023).

Now that most American 4‐year‐olds attend ECE, the focus of contemporary research has switched from comparing ECE attendance to home care and instead seeks to illuminate the relative benefits of different kinds of ECE programming (Joo et al. 2020). Correspondingly, researchers have called for long‐term follow‐up studies of rigorous randomized‐controlled trials (RCT) that compare different ECE components to better understand program characteristics that confer sustained benefits for children (Friedman‐Krauss et al. 2023; Joo et al. 2020; Watts et al. 2023). This study addressed this need by examining the high school outcomes of children who participated in the RCT of the Head Start Research‐based Developmentally Informed (REDI) program, which compared children attending Head Start classrooms enhanced with intensive social–emotional learning (SEL) supports (an evidence‐based SEL curriculum, synchronized SEL‐focused interactive reading program, professional development for teachers, and parent outreach materials) with children attending “usual practice” Head Start classrooms (Bierman et al. 2008). Research participants were followed longitudinally for 14 years from the start of preschool to the end of high school. Prior studies have documented that REDI promoted gains in emotional knowledge and social competence in preschool with benefits evident in social adjustment and parent involvement through elementary school (Bierman et al. 2008; Nix et al. 2013; Welsh et al. 2020). This study adds to those findings by reporting on late adolescent outcomes, including the primary outcomes of high school behavior problems and emotional symptoms and the secondary outcomes of high school GPA and on‐time graduation. In addition to assessing direct intervention effects on these outcomes, analyses included serial mediation path models to evaluate how earlier intervention effects contributed to later adolescent benefits. Identifying preschool intervention components that promote sustained benefits can inform future preschool policies and programming decisions, as well as contribute to models of social–emotional development and school adjustment.

### 1.1 ECE Programming Designed to Address Socioeconomic Disparities

Consistent with Head Start's goal of reducing socioeconomic disparities in school readiness and educational attainment, REDI intervention enhancements were designed to strengthen supports for children's early social–emotional development and language skills. Exposure to the adversities associated with poverty during early childhood has a negative impact on emerging social–emotional skills and associated school readiness (Blair and Raver 2015). Low‐income families have limited access to educational resources and experience chronic stressors that are often amplified by undesirable and unsafe living conditions (Evans and Kim 2013). Growing up under these unpredictable conditions can overwhelm the immature stress response systems of young children, impeding the development of executive functions and impulse control (Blair and Raver 2015). The result is a socioeconomic gap in the early development of the social–emotional, self‐regulatory, and language skills that support positive adjustment and learning as children progress through school (Bodovski and Youn 2011), thereby reducing educational attainment and increasing risk for adolescent externalizing and internalizing problems (Schreier and Chen 2013).

Enriching preschool classrooms with evidence‐based SEL programming has emerged as an effective strategy for boosting the social–emotional school readiness of children growing up in economically disadvantaged circumstances. Multiple meta‐analytic studies document the positive impact of preschool SEL programs on child social competence, emotional competence, behavioral self‐regulation, and reduced behavior problems (Blewitt et al. 2018; Luo et al. 2020; Murano et al. 2020; Schindler et al. 2015). Accordingly, the REDI program involved intensive SEL enrichment to Head Start classrooms designed to enhance teacher capacity to promote child social–emotional school readiness (Bierman et al. 2008). The foundation for REDI was an evidence‐based SEL program, Preschool PATHS (Domitrovich et al. 2007) that targeted prosocial skills, emotional understanding, and the self‐regulation of emotion and behavior. REDI also included a daily interactive reading program with book themes coordinated with PATHS to intensify SEL exposure and support oral language skill development, along with print centers and sound games to boost reading readiness. REDI coaches worked with teachers to promote positive classroom management practices and rich language use. Parents received materials illustrating REDI interaction strategies and home activity suggestions. By the end of the preschool year, REDI had significantly improved child emotion knowledge and social competence and boosted child vocabulary, print knowledge, and phonemic awareness (Bierman et al. 2008).

An important limitation of the research on preschool SEL is that very few studies have tracked preschool children over the transition into and through elementary school to determine whether preschool programming has longer‐term benefits. Existing longitudinal studies offer a mixed picture of the sustainability of preschool benefits, creating a need for research that explores the parameters and pathways associated with sustained gains.

### 1.2 Need to Understand Long‐Term Benefits of Randomized‐Controlled ECE Evaluations

The strongest evidence supporting the long‐term benefits of preschool education comes from studies that compared children who received intensive preschool intervention support with children who did not attend a preschool program (Burchinal et al. 2024). Contemporary studies of preschool interventions in which the control group includes children who attend preschool often show smaller benefits (Whitaker et al. 2023) with intervention gains that fade over time, typically becoming non‐significant within a few years after elementary school entry (Bailey et al. 2020; Claessens et al. 2014). Consistent with this pattern of effects, the gains in child vocabulary, print knowledge, and phonemic awareness that REDI promoted in preschool faded after the transition into elementary school (Bierman et al. 2014); in contrast, REDI social–emotional benefits were sustained through the end of elementary school (Welsh et al. 2020).

Researchers have speculated several reasons why the gains in social–emotional and self‐regulation skills promoted by preschool interventions may persist over time (Abenavoli 2019; Camilli et al. 2010). A central thesis is that boosts in social–emotional competencies and self‐regulation skills in childhood can set in motion positive developmental cascades by enhancing children's subsequent capacity to form positive relationships with teachers and peers and engage productively in classroom activities, thereby providing them with positive socialization supports and learning opportunities as they proceed through elementary school (Taylor et al. 2017). Conceptually, their enhanced preschool competencies foster more protective elementary school experiences that reduce their vulnerability to negative peer influences and school disengagement as they transition into adolescence (Taylor et al. 2017). Boosting preschool social–emotional and self‐regulation skills may thus foster sustained gains because they reduce risk for negative school experiences and improve the quality of proximal socialization supports—key processes that, in general, may underlie sustained preschool benefits (Bailey et al. 2020). This conceptual model has limited evidence to date as very few studies have followed children who received SEL interventions over time (Taylor et al. 2017).

Secondary analyses suggest that early boosts to preschool social–emotional skills may be the key factors producing the long‐term ECE effects on crime reduction and educational attainment found for the Perry Preschool project, Child Parent Center program, and Head Start (Deming 2009; Heckman et al. 2013; Reynolds and Ou 2011). However, to date, only one randomized‐controlled trial of a preschool SEL enhancement program, the Chicago School Readiness Program (CSRP) has followed participants through high school (Watts et al. 2023). CSRP focused on promoting Head Start teachers' capacity to manage classroom behavior in positive ways and providing teachers with supportive coaching designed to reduce their stress levels and promote their well‐being. Initial evaluations revealed significant benefits for children in the intervention classrooms, including gains in pre‐academic skills, executive functioning, and reduced behavior problems (Raver et al. 2011). Long‐term follow‐up during the initial high school years suggested sustained benefits in executive functioning and academic performance, although benefits faded by the end of high school (Watts et al. 2023). The evidence of sustained CSRP benefits through early adolescence provides some support for the speculation that preschool SEL boosts can initiate developmental cascades that promote adjustment across the adolescent transition, but also reflect a need for additional long‐term follow‐up studies of randomized preschool SEL intervention trials (Abenavoli 2019; Joo et al. 2020; Watts et al. 2023). There is also interest in understanding the nature of the developmental pathways that link preschool intervention gains to late adolescent benefits (Abenavoli 2019; Bailey et al. 2020; Pages et al. 2023).

### 1.3 The Present Study

The present study examined the late adolescent outcomes of youth who participated in the RCT of the REDI preschool program. Primary outcomes included externalizing (behavior problems) and internalizing (emotional symptoms) in grades 9 and 11, and secondary outcomes indexed academic success (high school GPA and on‐time graduation). In addition to confirmatory hypothesis‐testing evaluations of direct intervention effects on these outcomes, the study modeled exploratory serial mediation pathways to evaluate links between earlier intervention effects and the longer‐term high school outcomes. The hypothesis that intervention effects in preschool and elementary school would reduce adolescent adjustment problems and increase school success was based on the longitudinal logic model of SEL interventions articulated by Taylor and colleagues (2017). This model postulates that enhancing early social and emotional skills improves subsequent school experiences in middle childhood which prepare youth to cope adaptively with the challenges and opportunities associated with adolescence. Specifically, we hypothesized serial mediation pathways in which REDI would reduce high school emotional symptoms and behavior problems by boosting preschool emotion knowledge and social competence (Nix et al. 2013) and promoting grade school social adjustment and parent involvement (Welsh et al. 2020). We further explored the possibility that this serial mediation path would extend via reduced high school emotional symptoms and behavior problems to enhance school success (high school GPA and on‐time graduation). To test these hypotheses, we applied a multi‐phased mediation framework (Reynolds and Ou 2011).

## 2 Method

### 2.1 Procedures

In 2003 and 2004, participants were recruited at the start of their pre‐kindergarten year from 44 classrooms located in 26 Head Start centers in three Pennsylvania counties. Classrooms were stratified by county, program features (e.g., full day vs. half‐day), and population demographics (e.g., percent of children of color) and randomized to receive the REDI enhancements (intervention group) or serve as “usual practice” Head Start comparison classrooms (control group). Pre‐ and post‐intervention measures were collected in preschool. Children were followed longitudinally as they left Head Start and dispersed widely across schools, attending 202 kindergarten classrooms in 82 elementary schools in 33 school districts. Subsequent follow‐up assessments occurred in grade school (kindergarten and grades 1, 2, 3, 5) when each child's primary classroom teacher was asked to provide ratings of school adjustment. After students transitioned into high school (grades 9, 11), an academic subject (usually Language Arts) teacher was asked to provide ratings. School records were collected at the end of grade 12. Only Head Start teachers were aware of the intervention‐control status of the children they worked with; parents, observers, and subsequent grade school and high school teachers were naïve concerning intervention status.

The study complied with the ethical standards of the American Psychological Association, and methods were approved by the university Institutional Review Board. Written informed consent was obtained from parents, teachers, and youth who were age 18 and older; assent was obtained for youth under age 18. Teachers, parents, and youth were compensated financially for completing assessments.

### 2.2 Participants

Families of pre‐kindergarten‐age children in all participating classrooms were sent brochures describing the study; 86% agreed to participate in the study. Family recruitment materials were identical across the intervention and control conditions; brochures described a developmental study involving assessments of child progress during Head Start and after transition into elementary school to evaluate links between Head Start experiences and subsequent school adjustment.

Participants included 356 children with a mean age of 4.49 years old at the time of recruitment (SD = 0.31, range = 3.72–5.65) and their parents. Parents reported child race/ethnicity as 25% Black, 17% Latinx, 58% White, and child sex as 54% female, 46% male. Families were low income (median annual income of $15,000 at the time of recruitment; average income‐to‐needs ratio of 0.88). Parent education levels included less than high school (31%), high school graduation or GED (60%), some post‐high school training or education (7%), and a 4‐year college degree (2%). Over half of the families (60%) included two parents (married or living with a cohabitating partner) and the other (40%) were single parents. All parents reported speaking some English at home; 20% noted that they also spoke Spanish at home. Child assessments and youth interviews were conducted in English; parents who indicated greater comfort conversing in Spanish than in English were provided with Spanish‐speaking interviewers who read the survey questions in English and provided clarification or translation into Spanish as needed.

### 2.3 Head Start REDI Intervention

Participating Head Start classrooms all used a whole‐child curriculum (Creative Curriculum or High Scope) in alignment with Head Start regulations. In classrooms that were randomized to the intervention, the whole‐child curriculum was enriched with skill‐targeting curricular enhancements, whereas in classrooms randomized to the comparison group, the teachers continued their usual practices in implementing the whole‐child curriculum. The REDI enrichments included the Preschool PATHS curriculum (Domitrovich et al. 2007) which provided weekly lessons and extension activities designed to build children's emotional understanding, social skills, self‐control, and problem‐solving skills. REDI added a coordinated interactive reading program that used two books per week to extend classroom discussion of PATHS themes and strengthen children's oral language skills (Bierman et al. 2008). Additional REDI components included sound games (to build phonemic awareness) and alphabet centers (to build print knowledge). Teachers received 4 days of workshop training and weekly mentoring visits from REDI trainers to support high‐fidelity implementation and to promote positive teaching strategies and rich language use in the classroom. Teachers delivered most of the planned PATHS lessons and interactive reading sessions (*M* = 86%) and trainers rated implementation quality as “adequate” to “strong” (Bierman et al. 2008; Domitrovich et al. 2009). Parents received weekly PATHS handouts and 3 DVDs designed to illustrate the program and help them align support at home.

### 2.4 Measures

#### 2.4.1 Preschool Intervention Effects

Following procedures used in a prior study (Nix et al. 2013), preschool intervention effects on SEL were assessed in the domains of emotion knowledge and social–emotional competence. Two preschool measures tapped emotion knowledge: the *Assessment of Children's Emotion Skills* measured recognition of facial feeling displays shown in photographs (Schultz et al. 2004) and the *Emotion Recognition Questionnaire* (Ribordy et al. 1988) assessed the identification of feelings associated with different scenarios. These two measures were standardized within the sample and averaged at pre‐intervention (*r* = 0.36) and post‐intervention (*r* = 0.41). For analyses, the post‐intervention scores were regressed on pre‐intervention scores to represent preschool gains in emotion knowledge, and residuals were used in the process models to represent individual gains during the intervention year.

Preschool social competence was represented by ratings provided by parents (at pre‐intervention) and teachers and observers (at post‐intervention; see Nix et al. 2013). Trained research assistants conducted observations during small group play sessions held on two separate days outside of the classroom using a standard set of novel toys to equalize play opportunities across classrooms (for more details, see Bierman et al. 2008). Head Start teachers were aware of the intervention‐control group status of their classroom; parents and observers were naïve concerning the classroom condition. Each source (parents, teachers, observers) completed the *Social Competence Scale* (Conduct Problems Prevention Research Group [CPPRG] 1995) which included 13 items describing prosocial behaviors and emotion regulation (*α*_parents_ = 0.87, *α*_teachers_ = 0.94, *α*_observers_ = 0.88) and the *Authority Acceptance* scale of the *Teacher Observation of Child Adaptation‐Revised* (TOCA‐R; Werthamer‐Larsson et al. 1991) which included 7 seven items describing aggressive and disruptive behaviors (*α*_parents_ = 0.86, *α*_teachers_ = 0.95, *α*_observers_ = 0.92). Teacher aggression ratings included 6 additional items describing relational aggression. Observer aggression ratings were logged to adjust the positively skewed distribution. To create composite scores for analyses, each individual post‐intervention measure of social competence and aggression (reverse‐scored) provided by teachers and observers was standardized within the sample and then averaged (*α* = 0.70) and regressed on pre‐intervention parent ratings (also standardized and averaged, *r* = 0.67) to produce a residualized score representing preschool gains in social competence.

#### 2.4.2 Grade School Social Adjustment and Parent Involvement

Grade school teachers rated children on the *Social Competence Scale* (described above) in kindergarten (*α* = 0.95), grade 1 (*α* = 0.95), grade 2 (*α* = 0.94), grade 3 (*α* = 0.94), and grade 5 (*α* = 0.94). Grade school teachers also rated children on the *Student‐Teacher Relationship Scale*—*Closeness* (Pianta 2001) which included 8 items describing friendly, cooperative student‐teacher interactions rated on a 5‐point scale at each time point: kindergarten (*α* = 0.88), grade 1 (*α* = 0.89), grade 2 (*α* = 0.89), grade 3 (*α* = 0.90), and grade 5 (*α* = 0.91). In addition, grade school teachers rated children on the School Readiness Questionnaire (Bierman et al. 2008) with items describing adaptive approaches to learning and work habits, including 14 items in kindergarten (*α* = 0.97), grade 1 (*α* = 0.96), and grade 2 (*α* = 0.93) and a shorter 7‐item version scale in grade 3 (*α* = 0.90) and grade 5 (*α* = 0.94). Following procedures used in a prior study (Welsh et al. 2020) each of these three measures of grade school teacher ratings of social competence, student‐teacher closeness, and school readiness were standardized within the sample, and the standard scores were averaged at each data collection wave: kindergarten (*α* = 0.81), grade 1 (*α* = 0.79), grade 2 (*α* = 0.77), grade 3 (*α* = 0.75), and grade 5 (*α* = 0.74). Year‐to‐year correlations of these composite scores showed moderate stability, ranging from *r* = 0.49 to *r* = 0.55, with stability from kindergarten to grade 5, *r* = 0.49. Composite scores were averaged across years (*α* = 0.82) to represent grade school social adjustment.

Grade school teachers also completed the *Parent‐Teacher Involvement Questionnaire* (Kohl et al. 2000) which included 9 items describing positive parent involvement and support of the child's education: kindergarten (*α* = 0.94), grade 1 (*α* = 0.93), grade 2 (*α* = 0.92), grade 3 (*α* = 0.93), and grade 5 (*α* = 0.94). These scores were moderately stable, with year‐to‐year correlations ranging from *r* = 0.28 to *r* = 0.49, and stability from kindergarten to grade 5, *r* = 0.28. They were standardized within the sample each year and averaged across the grade school years to represent parent involvement during grade school (*α* = 0.75).

#### 2.4.3 Primary Outcomes of High School Behavior Problems and Emotional Symptoms

In high school (grades 9 and 11), youth and their parents each independently completed the *Report of School Adjustment—Discipline Scale* (CPPRG 1997) which included 3 items describing school behavior problems (e.g., getting into trouble with teachers, breaking school rules), youth grade 9 (*α* = 0.76) and grade 11 (*α* = 0.75), parent grade 9 (*α* = 0.90) and grade 11 (*α* = 0.86). An academic classroom teacher rated three items from the *TOCA‐R* (described above) describing the frequency of each participant's rule‐breaking, fighting, and non‐compliance in grade 9 (*α* = 0.93) and grade 11 (*α* = 0.86) with scores log‐transformed to normalize the positively skewed distributions. In addition, teachers completed the *Strength and Difficulties Questionnaire* (SDQ; Goodman 1999), including the 5‐item *Conduct Problems* scale (e.g., fights, lies or cheats, *α* = 0.84 grade 9, *α* = 0.77 grade 11). These teacher measures were composited to form one measure. Within rater (e.g., parent, youth, teacher), ratings were averaged over grades 9 and 11, and then each measure was standardized within the sample and composited. For analyses, high school behavior problems were represented by this multi‐rater composite (the average of youth, parent, and teacher ratings, *α* = 81).

Teacher ratings on the SDQ 5‐item *Emotional Symptoms* scale assessed internalizing problems (e.g., worried, unhappy, and depressed) in grade 9 (*α* = 0.78) and grade 11 (*α* = 0.81). Ratings were averaged across grades 9 and 11.

#### 2.4.4 Secondary Outcomes of High School GPA and On‐Time Graduation

Grades were obtained from school records collected at the end of high school. Grades were coded using a 13‐point scale (1 = F to 13 = A+) to create a uniform measure across the systems used by different school districts and then transformed to a 4‐point scale for ease of interpretation. Unweighted GPAs were calculated based on all high school grades available for each participant. On‐time graduation was represented by a dichotomous variable indicating whether the youth graduated on time, based on information collected from school records and youth interviews after the end of high school.

### 2.5 Baseline Equivalence Tests and Missing Data

*T*‐tests (for continuous variables) or *χ* ^2^ tests (for categorical variables) were conducted to evaluate any intervention‐control group differences at baseline. Groups were equivalent on most child demographics (e.g., age, sex, family SES, and proportions of White and Latinx children); however, the control group had a significantly higher proportion of Black children than the intervention group. No baseline group differences emerged on pretreatment scores for measures included in the longitudinal study, or proxy predictors of child school success (vocabulary and block design, see Table S2).

**FIGURE 1:**
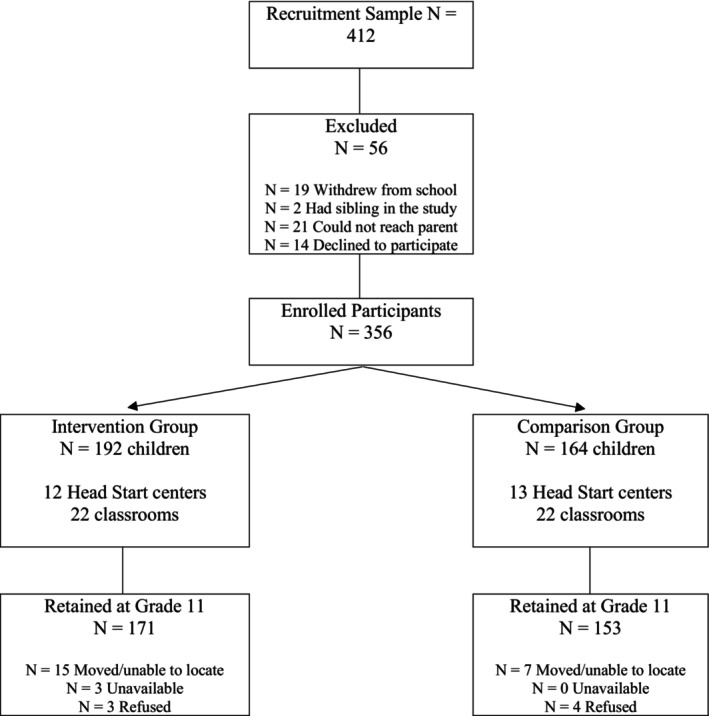
Consort diagram of participant flow through study.

There was intermittent missing data across the longitudinal waves of data collection, but few cases were lost to the study (see Table S3). At the grade 11 assessment, at least one of the raters (parent, youth, teacher) provided data for 324 youth, 91% of the original sample (see CONSORT diagram, Figure 1). Grade 11 data availability was comparable for children in the intervention and control conditions. *T*‐tests comparing participants for whom Grade 11 data were not available showed greater data availability for children who, at baseline, had higher scores on emotion knowledge, higher social competence, and higher family SES. School records providing data on GPA and graduation were available for 213 participants (60% of the original sample); post‐high school participants provided additional graduation data for 34 participants (total 69% of the original sample). *T*‐tests revealed that participants with GPA data had higher baseline vocabulary and child emotion knowledge scores than those without GPA data, and participants with graduation data had higher baseline vocabulary scores, higher family SES, and were more likely to be in the control group than the intervention group (see Tables S4 and S5). These analyses describing factors associated with intervention‐control group variation at baseline or attrition informed the selection of covariates, which included baseline scores on emotion knowledge (ACES, ERQ) and vocabulary, child race/ethnicity, and family SES. In addition, child age and sex were included as covariates based on their association with the adolescent outcomes included in this study, and county site was included to control for variations in the corresponding community characteristics as well as the county‐level organization of Head Start and school district programming.

Data were considered missing at random and taken into account by using multiple imputation (PROC MI; SAS 9.4) as recommended by Schafer and Graham (2002). 40 imputed data sets were created which included all individual measures mentioned above as well as the baseline covariates.

### 2.6 Plan for Analyses

Intervention effects on each high school outcome (behavior problems, emotional symptoms, GPA, on‐time graduation) were evaluated in separate hierarchical linear models (HLM) using SAS PROC MIXED for the continuous outcomes and PROC GLIMMIX for the binary outcome of on‐time graduation. When individual‐level outcomes are analyzed in the context of cluster randomized trials, it is important to appropriately adjust the standard errors to account for the dependencies that may exist among individuals in each cluster (Jacob et al. 2010; Spybook et al. 2020; What Works Clearing House 2022). The present study used a two‐level HLM model in which children (level 1) were nested in Head Start classrooms (level 2). PROC MIANALYZE was used to combine the results of analyses across the 40 imputed data sets. The formulas were given as follows:

$$\text{Level}\;1\;\left(\text{child}\right):Y_{\mathrm{ij}}=\beta_{0j}+\sum_{q=1}^{Q}\beta_{\mathrm{qj}}X_{\mathrm{qj}}+\gamma_{\mathrm{ij}},\;\gamma_{\mathrm{ij}}\sim N\left(0,\sigma^{2}\right)$$

$$\text{Level}\;2\;\left(\text{classroom}\right):\beta_{0j}=\gamma_{00}+\gamma_{01}{\left(\text{Intervention}\right)}_{j}+u_{0j},\;u_{0j}\sim N\left(0,\tau \right)$$

where $X_{\mathrm{qj}}$ represents subject‐level covariates as described in the methods portion of the text. The intervention effect is represented as a binary variable in the level 2 (Head Start classroom) level as $\gamma_{01}$. $\sigma^{2}$ and τ are the variance components for levels 1 and 2 respectively, conditional on all covariates.

Models of the primary outcomes (high school behavior problems, emotional symptoms) included serial mediators assessed at post‐intervention (e.g., residualized preschool gain scores of emotion knowledge and social competence) and over the course of grade school (social adjustment and parent involvement). Models of the secondary outcomes (GPA, on‐time graduation) included the same serial post‐intervention and grade school mediators along with high school mental health (behavior problems and emotional symptoms.) All models included the set of covariates designated above. All possible paths were estimated in the longitudinal path models (see Figure S1); additional figures show only the statistically significant pathways. To determine the significance of the mediation pathways, 1000 data sets were drawn in order to obtain appropriate bootstrapped confidence intervals as described by Wu et al. (2015). Analyses used SAS version 9.4 and Mplus 8.4 software.

## 3 Results

### 3.1 Preliminary Analyses

Means and standard deviations for composite variables are shown in Table S1. Correlations among the study variables are shown in Table 1. Residualized gains in the two preschool target skills (emotion knowledge, social competence) were significantly but only mildly correlated (*r* = 0.14), and both were significantly correlated with grade school social adjustment (*r*s = 0.18 and 0.37, respectively). Gains in preschool emotion knowledge were prospectively correlated with grade school parent involvement (*r* = 0.11) and on‐time graduation (*r* = 0.20); gains in preschool social competence were also prospectively correlated with reduced high school behavior problems (*r* = *−*0.19) and emotional symptoms (*r* = *−*0.18). Grade school social adjustment and parent involvement were similarly prospectively correlated with reduced high school behavior problems (*r*s = −0.34 and −0.25) and emotional symptoms (*r*s = −0.16 and −0.15) and with GPA (*r*s = 0.33 and 0.28); grade school social adjustment was also correlated with subsequent on‐time graduation (*r* = 0.24). In high school, behavior problems and emotional symptoms were linked prospectively with a lower likelihood of on‐time graduation (*r*s = −0.21 and −0.18) and behavior problems were also correlated with lower GPAs (*r* = *−*0.37).

**TABLE 1:** Correlations among study variables included in longitudinal models.

Variables	1	2	3	4	5	6	7
Change during head start							
1. Emotion knowledge							
2. Social competence	0.14*						
Grade school adjustment							
3. Social adjustment	0.18**	0.37***					
4. Parent involvement	0.11*	0.01	0.32***				
High school problems—Primary outcomes							
5. Behavior problems	−0.04	−0.19**	−0.34***	−0.25***			
6. Emotional symptoms	0.02	−0.18**	−0.16*	−0.15*	0.38***		
Academic outcomes—Secondary outcomes							
7. Grade point average	0.08	0.12	0.33***	0.28***	−0.37***	−0.10	
8. On‐time graduation	0.20**	0.08	0.24***	0.10	−0.21**	−0.18**	0.09

* *p* < 0.05.

** *p* < 0.01.

*** *p* < 0.001.

### 3.2 Intervention Effects

Hierarchical linear models revealed that children who received the REDI preschool program during Head Start had significantly fewer high school behavior problems (estimate = −0.23, SE = 0.10, *p* = 0.03) and emotional symptoms (estimate = −0.71, SE = 0.21, *p* < 0.001) than children in the control group (see Table 2). Outcome variables were standardized for analyses, creating estimates that are equivalent to effect sizes (Cohen's *d*), thus representing an effect of *d* = 0.27 on behavior problems and an effect of *d* = 0.41 on emotional symptoms. Hierarchical linear models revealed non‐significant intervention effects on the secondary outcomes of high school GPA (estimate = 0.10, SE = 0.15, *p* = 0.49) and on‐time graduation (estimate = 0.50, SE = 0.41, *p* = 0.23).

**TABLE 2:** HLM tests of direct intervention effects on high school outcomes.

	Intervention group		Control group		Intervention effect		
	Mean	SE	Mean	SE	Estimate	SE	*p*
Behavior problems	−0.16	0.78	0.08	0.94	−0.23	0.10	0.03*
Emotional symptoms	1.51	1.41	2.18	1.98	−0.71	0.21	0.001**
GPA	1.80	1.31	1.66	1.21	0.10	0.15	0.49
On‐time graduation	0.90	0.30	0.85	0.36	0.50	0.41	0.23

Abbreviation: SE, standard error.

* *p <* 0.05.

** *p <* 0.01.

### 3.3 Indirect Paths of Intervention Influence—Primary Outcomes

**FIGURE 2:**
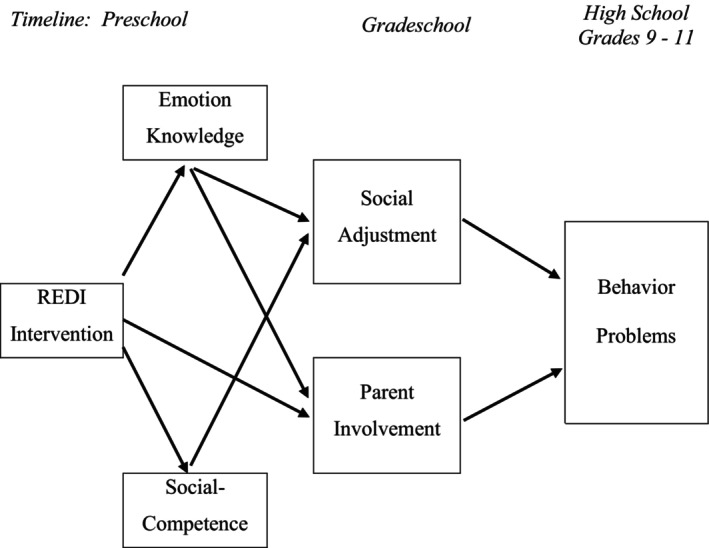
Significant paths linking preschool intervention to high school behavior problems. This figure illustrates the four significant indirect intervention paths to reduced behavior problems based upon bootstrapped confidence intervals; specific path coefficients are shown in Table S6. The paths included: Intervention → Emotion Knowledge → Social Adjustment → Behavior Problems; Intervention → Emotion Knowledge → Parent Involvement → Behavior Problems; Intervention → Social Competence → Social Adjustment → Behavior Problems; Intervention → Parent Involvement → Behavior Problems. Paths that did not contribute to significant mediation are not shown.

To better understand how REDI produced long‐term benefits, the first serial path model examined intervention‐related gains in preschool emotion knowledge and social competence and subsequent grade school social adjustment and parent involvement as mediators of intervention effects on high school behavior problems. Four significant mediation paths emerged (see Figure 2, Table S6). Two significant pathways began with intervention boosts in preschool emotion knowledge, which then progressed through grade school social adjustment and parent involvement to reduced behavior problems. Another significant mediation path began with preschool gains in social competence and cascaded through grade school social adjustment to reduced behavior problems. The final path extended from REDI to enhanced parent involvement, which led to reduced behavior problems. Taken together, these four paths highlight the importance of the REDI program impact on child social–emotional cognitions and behaviors as well as on parent involvement as key foundations promoting longer‐term mental health benefits.

**FIGURE 3:**
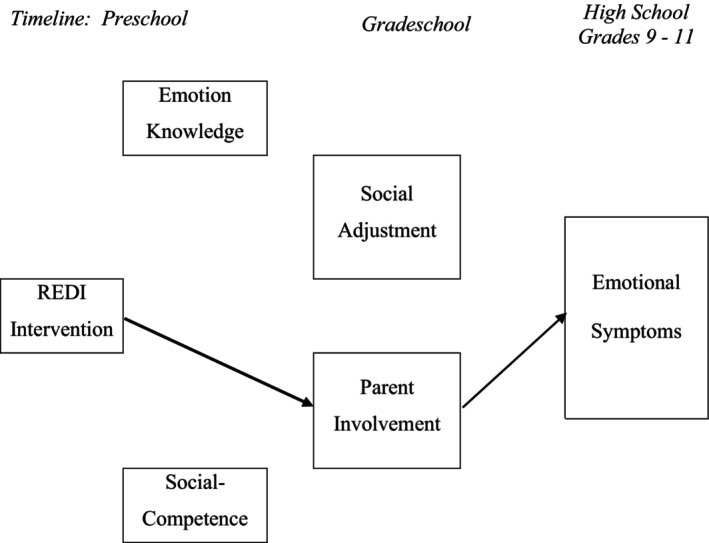
Significant paths linking preschool intervention to high school emotional symptoms. This figure illustrates the significant indirect intervention path to reduced emotional symptoms based upon bootstrapped confidence intervals; specific path coefficients are shown in Table S7. The path included Intervention → Parent Involvement → Emotional Symptoms. Paths that did not contribute to significant mediation are not shown.

The second model tested mediation pathways from the preschool intervention to high school emotional symptoms. One significant mediated pathway emerged (Figure 3, Table S7), progressing from intervention effects on parent involvement to reduced high school emotional symptoms. Thus, REDI's promotion of parent involvement figured centrally in improving adolescent emotional well‐being.

### 3.4 Indirect Paths of Intervention Influence—Secondary Outcomes

**FIGURE 4:**
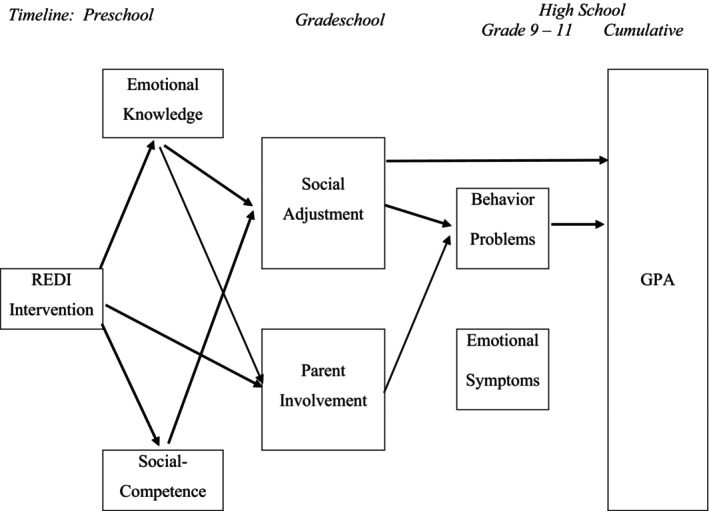
Significant paths linking preschool intervention to cumulative high school GPA. This figure illustrates the 6 significant indirect intervention paths to improved GPA based upon bootstrapped confidence intervals; specific path coefficients are shown in Table S8. The paths extend from the 4 predicting reduced Behavior Problems (see Table S6, Figure 2): Intervention → Emotion Knowledge → Social Adjustment → Behavior Problems → GPA; Intervention → Emotion Knowledge → Parent Involvement → Behavior Problems → GPA; Intervention → Social Competence → Social Adjustment → Behavior Problems → GPA; Intervention → Parent Involvement → Behavior Problems → GPA. There were also two additional paths that did not include behavior problems: Intervention → Emotion Knowledge → Social Adjustment → GPA; Intervention → Social Competence → Social Adjustment → GPA. Paths that did not contribute to significant mediation are not shown.

Next, we examined the longitudinal path models for each of the secondary outcomes including high school behavior problems and emotional symptoms as potential mediators along with preschool intervention effects and grade school functioning. The model predicting high school GPA revealed six statistically significant mediation paths (see Figure 4, Table S8). Each of the four paths that mediated intervention effects on reduced high school behavior problems (Figure 2) also cascaded to improved GPA, extending from gains in preschool emotion knowledge and social competence via grade school social adjustment and parent involvement. Two additional paths extended from intervention‐related boosts in grade school adjustment to improved GPA—one through preschool gains in emotion knowledge and one direct from REDI intervention to grade school adjustment. These paths highlight the indirect ways in which the REDI intervention enhanced GPA: via enhancements to initial social–emotional competencies that promoted grade school social adjustment and reduced high school behavior problems.

**FIGURE 5:**
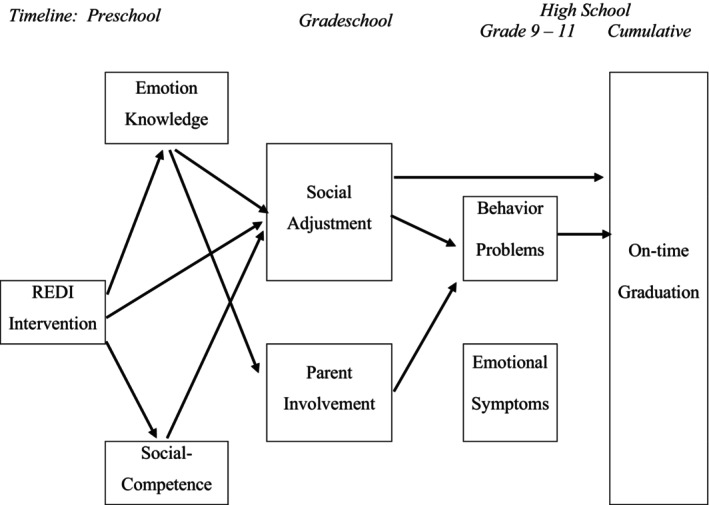
Significant paths linking preschool intervention to on‐time graduation. This figure illustrates the 6 significant indirect intervention paths to On‐time Graduation based upon bootstrapped confidence intervals; specific path coefficients are shown in Table S9. The paths include 3 that extended from reduced Behavior Problems (see Table S6, Figure 2): Intervention → Emotion Knowledge → Social Adjustment → Behavior Problems → Graduation; Intervention → Emotion Knowledge → Parent Involvement → Behavior Problems → Graduation; Intervention → Social Competence → Social Adjustment → Behavior Problems → Graduation. There were two additional paths through grade school social adjustment: Intervention → Emotion Knowledge → Social Adjustment → Graduation, Intervention → Social Adjustment → Graduation, and an additional path through Parent Involvement: Intervention → Parent Involvement → Behavior Problems → Graduation. Paths that did not contribute to significant mediation are not shown.

The final model considered indirect paths from preschool intervention to on‐time graduation and revealed six significant mediation paths (Figure 5, Table S9). The four pathways that mediated intervention effects on reduced high school behavior problems extended to significantly mediate increases in on‐time graduation (just as they also extended to mediate improvements in GPA). In addition, two significant mediation paths involved grade school social adjustment, one that extended from REDI growth in preschool emotion knowledge to improved grade school social adjustment to increases in on‐time graduation and one that extended directly from REDI to improved grade school social adjustment to increases in on‐time graduation.

## 4 Discussion

The REDI program enhanced Head Start classrooms with an evidence‐based SEL curriculum (Preschool PATHS) and an aligned daily interactive reading program that extended discussions on social–emotional themes. Teachers received coaching in interaction strategies to strengthen social–emotional supports (positive management, emotion coaching, problem‐solving dialogue). Parents received weekly handouts and three DVDs illustrating program strategies that could be used at home. Compared with children randomized to usual practice Head Start classrooms, those who got the REDI enrichments showed significantly fewer behavior problems and emotional symptoms in high school—14 years after the intervention. Path analyses revealed that these late adolescent mental health benefits emerged as a function of positive cascades initiated by intervention‐related gains in preschool emotion knowledge and social competence that then enhanced parent involvement and social adjustment during grade school. Reductions in high school behavior problems mediated indirect intervention effects on academic attainment—GPA and on‐time graduation. Interestingly, additional REDI effects were evident on grade school parent involvement and social adjustment (beyond the intervention effects mediated by preschool gains) which also contributed indirectly to later academic attainment.

The study findings show that leveraging existing systems of ECE delivery to promote student social–emotional skills and family involvement offers an important strategy for enhancing the adolescent mental health and school adjustment of children growing up in poverty. Efforts to promote the well‐being of youth growing up in vulnerable circumstances are especially urgent following the COVID‐19 pandemic, due to increases in child poverty and widened socioeconomic disparities in academic achievement and well‐being (Gee et al. 2023; Vaalavuo et al. 2022).

### 4.1 The Lasting Effects of Intensive SEL Support in ECE

REDI provided an intensive boost to preschool SEL with coordinated curriculum, interactive reading, and teacher coaching. REDI also included print centers and sound games to promote letter knowledge and phonemic awareness, but initial intervention effects on literacy skills (vocabulary, print awareness, phonemic awareness) faded quickly after children entered grade school (Bierman et al. 2008, 2014). The immediate benefits of ECE enrichment often fade out as children move through grade school, when children in the control group begin to catch up with formal instruction and those who received preschool intervention show a reduced pace of skill acquisition, possibly due to a lack of aligned instruction that builds on preschool gains (Burchinal et al. 2024; Whitaker et al. 2023). Researchers have speculated that ECE‐promoted gains in social–emotional skills may have more durability than commonly targeted academic skills because they increase child social and academic engagement at school entry, promoting the capacity to benefit from grade school instruction (Abenavoli 2019; Camilli et al. 2010). In addition, elementary schools often lack systematic SEL instruction which may reduce the catch‐up of children in the control group (Abenavoli 2019; Pages et al. 2023). However, there is very little research examining the longitudinal impact of preschool SEL programs. The CSRP is the only prior study that has evaluated the impact of a preschool SEL program on high school outcomes in the context of a rigorous randomized‐controlled design, documenting evidence of some sustained benefits in the initial high school years that faded by the end of high school (Watts et al. 2023). The CSRP and REDI interventions both involved enrichments to Head Start classrooms and used randomized designs with “usual practice” Head Start classrooms serving as the comparison group. However, the programs differed in intervention focus, as REDI emphasized curricular enrichments and CSRP emphasized teacher professional development and support. In addition, the community contexts varied substantially, as CSRP Head Start centers were located in a large, densely populated urban area (Chicago) and REDI Head Start centers were spread across three Pennsylvania counties that encompassed smaller urban communities along with rural areas and small towns. Both studies offer evidence that preschool classroom SEL enrichments can produce long‐term benefits for children, but additional research is needed to better understand the mediating processes that account for sustained effects and the factors that influence fade out of benefits over time.

Researchers have speculated that the long‐term effects of ECE often reflect cascading indirect effects over time rather than the stable sustainability of initial benefits (Pages et al. 2023; Reynolds and Ou 2011). In cascade models, early skill‐boosting assists children in successfully tackling subsequent developmental challenges (Sandler et al. 2015) or accessing more protective socialization contexts (Pages et al. 2023) that in turn promote subsequent benefits in school adjustment and functioning. For example, Reynolds and Ou (2011) showed that the Child Parent Center program produced initial gains in child cognitive skills and family engagement, which enhanced grade school behavior, parent involvement, and academic performance, which in turn promoted better school placement, reduced juvenile arrest, and increased rates of high school completion. Progressive indirect effects also emerged in the present study, as high school outcomes were mediated by preschool intervention effects cascading via multifaceted developmental pathways. Normatively, the transition into adolescence is accompanied by a host of new developmental challenges (Jacobson et al. 2011) as youth navigate larger and more complex social networks at school, face higher academic demands, and have more freedom to make decisions about their time use (Moody et al. 2011). These shifts in school contexts and social influences, along with pubertal psychophysiology, contribute to heightened levels of impulsive risk‐taking, rule‐breaking, and emotional distress (Steinberg et al. 2018). By increasing early social–emotional skills and boosting grade school social support from teachers and parents, REDI likely facilitated adolescents' capacity to successfully navigate these challenges and thereby reduced adolescent behavior problems and emotional symptoms which, in turn, fostered better academic outcomes.

It is important to note that REDI included intensive exposure to SEL (SEL curriculum, integrated interactive reading, teacher coaching). The intervention increased the frequency and quality of teacher language use in the classroom and enhanced teacher attention to emotions and proactive classroom management (Domitrovich et al. 2009). The intensity of the SEL focus along with the integrated support for child language development may have been key to its long‐term effects. In a meta‐analysis of ECE program evaluations, Joo et al. (2020) concluded that the use of evidence‐based curricula increased the impact of ECE programs on child school readiness beyond the use of teacher professional development supports alone, possibly because these programs support teachers in providing daily systematic support for child skill acquisition. Intensive SEL such as that provided by REDI may be especially beneficial for preschool children who have experienced poverty in early childhood given the negative impact of associated adversity on social–emotional development (Blair and Raver 2015).

### 4.2 Improving Adolescent Mental Health and Behavioral Adjustment

The REDI intervention impact on high school behavior problems was *d* = 0.23, reflecting a mean reduction of about one‐quarter of a standard deviation for children who received the REDI enrichment during Head Start compared to those who experienced usual practice Head Start. The corresponding impact on high school emotional symptoms was even stronger, *d* = 0.41. These effects are notable for a universal educational intervention delivered at the classroom level (Kraft 2020), particularly considering the time lag between the preschool intervention and these high school outcome assessments. These effects may confer additional benefits in the future by reducing risk for substance use, interpersonal difficulties, psychological distress, and underemployment in adulthood (McLeod et al. 2016; Veldman et al. 2015).

REDI did not have a significant direct impact on high school GPA or on‐time graduation, but the significant mediated pathways from intervention to each of those outcomes suggest systematic influence. Given the lack of statistical significance, additional follow‐up is needed to determine whether the indirect effects will result in subsequent positive outcomes for youth self‐sufficiency, employment and earnings, and civic engagement, which are related to high school GPA (French et al. 2015) and on‐time graduation (Zaff et al. 2017).

### 4.3 Study Strengths and Limitations

Study strengths included a relatively large sample size, long‐term prospective longitudinal design, and randomized‐controlled trial that allowed for unbiased estimates of intervention impact. In addition, measures included multiple reporters and multi‐year composites that likely increased the precision of measurement.

One important limitation was that the sample included Head Start programs in three counties in Pennsylvania, which may not be representative of the programming and populations served by Head Start in other regions of the country. The sample was diverse but did not include sufficiently large subsamples to determine whether demographic or ethnic/cultural factors influenced response to intervention. In addition, participating families were low‐income, leaving unanswered questions about the impact of the intervention on children and families who represent a greater socioeconomic range. Rates of missing data were quite high for some outcome measures (31% missing data about on‐time graduation; 40% missing high school GPA) and the level of missing data was higher in the intervention than the control group. Analytic models adjusted for missing data, but the amount and nature of the differential missingness in these outcomes represent a study limitation. Finally, the REDI intervention was multi‐component, limiting the degree to which the current findings can be attributed to specific intervention components. We speculate that the daily curricular emphasis on SEL content via Preschool PATHS and a coordinated interactive reading program played a key role in promoting the gains in child emotion knowledge that served as one mediator of later benefits and that the addition of teacher coaching and parent outreach boosted gains in child social competence and parent‐teacher involvement that also served as mediators of later benefits. However, specific links between program components and intervention gains remain speculative.

### 4.4 Implications

The study findings extend prior follow‐up studies of the REDI program through the end of high school and identify significant positive developmental progressions that illuminate how the intervention achieved long‐term effects. There are important implications for educational policy and practice. Support for the expansion of publicly funded pre‐kindergarten has grown substantially in the United States. The findings suggest that these public investments could be leveraged by enriching the quality of programming, especially focusing more systematically on curriculum components and professional development to support integrated SEL and language development supports. The cost of REDI delivery averaged $191 per child (Jones et al. 2019) which is a small amount relative to the cost per child to fund Head Start and pre‐kindergarten programming. Additional follow‐up research is needed to determine whether these high school effects emerge as benefits that outweigh the initial social costs of the intervention and to determine the generalizability of these effects across different populations with variations in programming and delivery method. The current findings offer promising benefits that are worth additional exploration and extension.

## Disclosure

The views expressed in this article are ours and do not necessarily represent the granting agencies. Follow‐up analyses were registered in the Registry of Efficacy and Effectiveness Studies #16840.

## Conflicts of Interest

The authors declare no conflicts of interest.

## Supporting information


Data S1.

## Data Availability

The data necessary to reproduce the analyses presented here are not publicly accessible; requests for data can be made to the corresponding author. The analytic code necessary to reproduce the analyses will be made available upon request.
