# Supplementary material for: How a Preschool Intervention Affected High School Outcomes: Longitudinal Pathways in a Randomized‐Controlled Trial
Source: Child Dev. 2025 Apr 1;96(3):1236–49. doi: 10.1111/cdev.14235 (PMC12023813; doi:10.1111/cdev.14235)
Supplement: Supplementary file 1 — Data S1. [file CDEV-96-1236-s001.docx]

**Table S1**.

*Measures Used in Study Composite Constructs*

| Construct | Composited Measures | N | Control  Mean  (SD) | Intervention Mean  (SD) |
| --- | --- | --- | --- | --- |
| Preschool: Residualized Gain Scores | |  |  |  |
| Emotion Knowledge | *Assessment of Children’s Emotion Skills* (Schultz et al., 2004) - C  *Emotion Recognition Questionnaire* (Ribordy et al., 1988) - C | 335 | -0.19  (1.02) | 0.16  (0.96) |
| Social Competence | *Social Competence Scale* (CPPRG, 1995) – T, P, O  *Authority Acceptance*, TOCA-R (Werthamer-Larsson et al., 1991) – T, P, O  *Preschool Social Behavior Scale – Teacher* (Crick et al., 1997) - T | 345 | -0.16  (1.06) | 0.14  (0.93) |
| Gradeschool Mediators | |  |  |  |
| Social Adjustment | *Social Competence Scale* (CPPRG, 1995) - T  *School Readiness Questionnaire* (Bierman et al., 2008) - T  *Student-Teacher Relationship Scale – Closeness* (Pianta, 2001) - T | 336 | 4.44  (0.64) | 4.60  (0.64) |
| Parent Involvement | *Parent-Teacher Involvement Questionnaire* (Kohl et al., 2000) - T | 336 | 3.35  (0.81) | 3.56  (0.81) |
| High School (Primary Outcomes) | |  |  |  |
| Behavior Problems | *Report of School Adjustment* - *Discipline Scale* (CPPRG, 1997) – C, P  *Authority Acceptance (*TOCA-R; Werthamer-Larsson et al., 1991) – T  *SDQ Conduct Problems* (Goodman, 1999) - T | 289 |  |  |
| Emotional Symptoms | *SDQ Emotional Symptoms* (Goodman, 1999) *-* T | 259 | 2.21  (2.27) | 1.48  (1.93) |
| School Success (Secondary Outcomes) | |  |  |  |
| GPA | High School GPA | 213 | 1.73  (1.21) | 1.90  (1.30) |
| On-time Graduation | Dichotomous indicator – Graduated with cohort | 248 | 0.88  (0.33) | 0.90  (0.30) |

Note: C= child source, P = parent source, T = teacher source. N represents the number of participants with data available for measures used in the composite. Analyses were completed using multiple imputation to address missing data. Preschool measures were standardized within the sample, averaged into composites, and the values shown represent the residualized change over the course of the preschool year. Gradeschool measures were standardized within the sample and averaged into composites. Adolescent ratings of behavior problems were also standardized within the sample and averaged into composites. GPA was scored on a 13-point scale, with 1 = F, 6 = C, and 13 = A+. but for ease of interpretation, GPA mean scores are reported after conversion to the more familiar 4-point scale.

**Table S2.**

*Evaluating Baseline Equivalence for Participants in the Intervention and Control Samples*

| Baseline Scores | Intervention Group  (N = 193) | | Control Group  (N = 164) | | Group Differences | |
| --- | --- | --- | --- | --- | --- | --- |
|  | Mean | (SD) | Mean | (SD) | T-test | Effect size |
| Child Demographics |  |  |  |  |  |  |
| Age | 4.49 | (0.31) | 4.49 | (0.30) | 0.09 | 0.01 |
| Sex: Female | 52.06% | -- | 47.94% | -- | 0.60 | -- |
| Sex: Male | 56.17% | -- | 43.83% | -- | 0.60 | -- |
| Ethnicity/Race: White | 55.98% | -- | 44.02% | -- | 0.85 | -- |
| Ethnicity/Race: Black | 43.68% | -- | 56.32% | -- | 4.87* | -- |
| Ethnicity/Race: Latinx | 59.09% | -- | 40.91% | -- | 0.87 | -- |
| Family SES | 22.42 | (9.75) | 21.47 | (10.17) | -0.90 | 0.10 |
| Baseline Scores |  |  |  |  |  |  |
| Emotion Knowledge -ACES | 5.40 | (2.16) | 5.68 | (2.27) | 1.20 | 0.13 |
| Emotion Knowledge - ERQ | 1.37 | (0.31) | 1.34 | (0.29) | -1.04 | 0.11 |
| Vocabulary - EOWPVT | 34.91 | (11.12) | 34.62 | (10.76) | -0.24 | 0.03 |
| Block Design | 19.33 | (3.95) | 19.40 | (3.09) | 0.20 | 0.02 |
| Social Competence | 3.59 | (0.83) | 3.59 | (0.76) | 0.05 | 0.01 |
| Authority Acceptance | 2.88 | (1.02) | 2.93 | (0.94) | 0.45 | .05 |

Note: Baseline equivalence was evaluated for child demographics and for pretreatment scores on measures included in the longitudinal study (ACES, ERQ, social competence, externalizing behaviors) and proxy predictors of child school success (vocabulary and block design). Dichotomous variables (indicated with percentages) were evaluated using χ^2^ tests and continuous variables were evaluated with t-tests. * *p* < .05.

**Table S3.**

*Availability of Data (Teacher Data; Any Source of Data) At Each Grade Level Studied*

| Grade Level | Data from Teacher | | Data from Any Source  (Teacher, Parent, or Child) | |
| --- | --- | --- | --- | --- |
|  | N | (%) | N | (%) |
| Kindergarten | 321 | 90% | 337 | 95% |
| Grade 1 | 325 | 91% | 330 | 93% |
| Grade 2 | 302 | 85% | 314 | 88% |
| Grade 3 | 288 | 81% | 323 | 91% |
| Grade 5 | 264 | 74% | 286 | 80% |
| Grade 9 | 229 | 64% | 282 | 79% |
| Grade 11 | 193 | 54% | 324 | 91% |

Note: In the present study, teacher ratings were used to assess elementary school social adjustment and parent involvement and high school emotional symptoms. A composite of teacher, youth, and parent ratings was used to assess high school behavior problems.

**Table S4.**

*Participants Missing School Success Outcomes in the Intervention and Control Samples*

| School Success Outcome | Missing in  Intervention Group | | Missing in  Control Group | | Group Differences | |
| --- | --- | --- | --- | --- | --- | --- |
|  | Number | (%) | Number | (%) | χ^2^-test | p-value |
| GPA | 81 | (42%) | 62 | (38%) | 0.71 | 0.40 |
| On-time Graduation | 74 | (39%) | 44 | (27%) | 5.64 | 0.02 |

Note: This table shows the results of χ^2^ tests conducted to determine whether levels of missing data for the secondary high school outcomes of GPA and on-time graduation varied significantly for participants in the intervention versus control group.

**Table S5.**

*Baseline Predictors of Missingness in School Success Outcomes*

|  | GPA | | | | On-time Graduation | | | |
| --- | --- | --- | --- | --- | --- | --- | --- | --- |
|  | Missing | Retained | Test | p-value | Missing | Retained | Test | p-value |
| Child Demographics |  |  |  |  |  |  |  |  |
| Age | 4.50 | 4.49 | 0.50 | 0.62 | 4.50 | 4.49 | 0.36 | 0.72 |
| Sex: Female | 41% | 59% | 0.20 | 0.65 | 35% | 65% | 0.76 | 0.38 |
| Sex: Male | 39% | 61% | 0.20 | 0.65 | 31% | 69% | 0.76 | 0.38 |
| Ethnicity/Race: White | 37% | 63% | 3.05 | 0.08 | 31% | 69% | 0.90 | 0.34 |
| Ethnicity/Race: Black | 46% | 54% | 1.62 | 0.20 | 33% | 67% | 0.00 | 0.98 |
| Ethnicity/Race: Latinx | 48% | 52% | 2.33 | 0.13 | 38% | 62% | 0.79 | 0.38 |
| Family SES | 21.97 | 21.99 | -0.02 | 0.988 | 20.31 | 22.82 | -2.25* | 0.03 |
| Baseline Scores | |  |  |  |  |  |  |  |
| Emotion Knowledge -ACES | 5.15 | 5.79 | -2.66* | 0.001 | 5.28 | 5.65 | -1.49 | 0.14 |
| Emotion Knowledge-ERQ | 1.30 | 1.40 | -03.02* | 0.003 | 1.34 | 1.37 | -0.89 | 0.37 |
| Vocabulary - EOWPVT | 32.40 | 36.37 | -3.39* | < .001 | 32.78 | 35.77 | -2.43* | 0.02 |
| Block Design | 19.26 | 19.43 | -0.46 | 0.65 | 19.15 | 19.47 | -0.77 | 0.44 |
| Social Competence | 3.58 | 3.60 | -0.15 | 0.88 | 3.63 | 3.57 | 0.62 | 0.54 |
| Authority Acceptance | 3.01 | 2.83 | 1.63 | 0.10 | 2.99 | 2.86 | 1.15 | 0.25 |

Note: This table examined possible associations between baseline variables and later missing data for GPA and on-time graduation. Baseline variables included child demographics, pretreatment scores on measures included in the longitudinal study (ACES, ERQ, social competence, authority acceptance), and proxy predictors of child school success (vocabulary and block design). * *p* < .05.

**Table S6.**

*Serial Longitudinal Path Model Exploring Mediation of High School Behavior Problems*

| Preschool | Gradeschool | High School | *Estimate* | 95% CI |
| --- | --- | --- | --- | --- |
| 3-step Mediation Paths | | | | |
| INT -> Emotion Knowledge -> | Social Adjustment -> | Behavior Problems | -.012 | -.032, -.003* |
| INT -> Emotion Knowledge -> | Parent Involvement -> | Behavior Problems | -.006 | -.021, -.000* |
| INT -> Social Competence -> | Social Adjustment -> | Behavior Problems | -.024 | -.056, -.007* |
| INT -> Social Competence -> | Parent Involvement -> | Behavior Problems | .002 | -.002, .014 |
| 2-step Mediation Paths (Without Preschool Effects) | | | | |
| INT -> | Social Adjustment -> | Behavior Problems | -.022 | -.091, .026 |
| INT -> | Parent Involvement -> | Behavior Problems | -.043 | -.110, -.004* |
| 2-step Mediation Paths (Without Gradeschool) | | | | |
| INT-> Emotional Knowledge-> |  | Behavior Problems | .013 | -.025, .060 |
| INT -> Social Competence -> |  | Behavior Problems | -.020 | -.080, .010 |

Note: INT = preschool intervention. Preschool measures (emotion knowledge, social competence) were standardized within the sample, averaged into composites, and the values shown represent the residualized change over the course of the preschool year. Gradeschool measures (teacher-rated social adjustment, parent involvement) were standardized within the sample and averaged into composites. High school ratings of behavior problems by parents, teachers, and youth were standardized within the sample and averaged into composites. * *p <* .05

**Table S7.**

*Serial Longitudinal Path Model Exploring Mediation of High School Emotional Symptoms*

| Preschool | Gradeschool | High School | *Estimate* | 95% CI |
| --- | --- | --- | --- | --- |
| 3-step Mediation Paths | | | | |
| INT -> Emotion Knowledge -> | Social Adjustment -> | Emotional Symptoms | -.001 | -.013, .005 |
| INT -> Emotion Knowledge -> | Parent Involvement -> | Emotional Symptoms | -.004 | -.017, .000 |
| INT -> Social Competence -> | Social Adjustment -> | Emotional Symptoms | -.003 | -.022, .011 |
| INT -> Social Competence -> | Parent Involvement -> | Emotional Symptoms | .001 | -.002, .012 |
| 3-step Mediation Paths (Without Preschool Effects) | | | | |
| INT -> | Social Adjustment -> | Emotional Symptoms | -.003 | -.041, .010 |
| INT -> | Parent Involvement -> | Emotional Symptoms | -.030 | -.094, -.000* |
| 2-step Mediation Paths (Without Gradeschool) | | | | |
| INT-> Emotional Knowledge-> |  | Emotional Symptoms | .034 | -.005 .102 |
| INT -> Social Competence -> |  | Emotional Symptoms | -.034 | -.102, .001 |

Note: INT = preschool intervention. Preschool measures (emotion knowledge, social competence) were standardized within the sample, averaged into composites, and the values shown represent the residualized change over the course of the preschool year. Gradeschool measures (teacher-rated social adjustment, parent involvement) were standardized within the sample and averaged into composites. High school teacher ratings of emotional symptoms were standardized within the sample and averaged across grades. * *p <* .05

* *p <* .05

**Table S8.**

| Preschool | Gradeschool | High School | *Est.* | 95% CI |
| --- | --- | --- | --- | --- |
| Full 4-step Mediation Paths | | | | |
| INT -> Emotion Knowledge-> | Social Adjustment -> | Behavior Problems -> GPA | .003 | .001, .010* |
| INT -> Emotion Knowledge-> | Parent Involvement -> | Behavior Problems -> GPA | .001 | .000, .006* |
| INT -> Social Competence -> | Social Adjustment -> | Behavior Problems -> GPA | .006 | .002, .016* |
| INT -> Social Competence -> | Parent Involvement -> | Behavior Problems -> GPA | -.000 | -.004, .001 |
| INT -> Emotion Knowledge-> | Social Adjustment -> | Emotional Symptoms-> GPA | -.000 | -.002, .000 |
| INT -> Emotion Knowledge-> | Parent Involvement -> | Emotional Symptoms-> GPA | -.000 | -.001, .000 |
| INT -> Social Competence -> | Social Adjustment -> | Emotional Symptoms-> GPA | -.000 | -.003, .000 |
| INT -> Social Competence -> | Parent Involvement -> | Emotional Symptoms-> GPA | .000 | -.000, .001 |
| 3-step Mediation Paths (Without High School Behavior Problems) | | | | |
| INT -> Emotion Knowledge-> | Social Adjustment -> | GPA | .010 | .002, .032* |
| INT -> Emotion Knowledge-> | Parent Involvement-> | GPA | .004 | -.000, .018 |
| INT -> Social Competence -> | Social Adjustment -> | GPA | .020 | .005, .048* |
| INT -> Social Competence -> | Parent Involvement -> | GPA | -.001 | -.011, .002 |
| 3-step Mediation Paths (Without Preschool Effects) | | | | |
| INT -> | Social Adjustment -> | Behavior Problems -> GPA | .005 | -.006, .025 |
| INT -> | Parent Involvement -> | Behavior Problems -> GPA | .011 | .001, .031* |
| INT -> | Social Adjustment -> | Emotional Symptoms-> GPA | -.000 | -.007, .000 |
| INT -> | Parent Involvement -> | Emotional Symptoms-> GPA | -.001 | -.011, .001 |
| 2-step Mediation Paths (Without Behavior Problems or Preschool Effects) | | | | |
| INT -> | Social Adjustment -> | GPA | .019 | -.021, .078 |
| INT -> | Parent Involvement -> | GPA | .026 | -.001, .090 |

*Serial Longitudinal Path Model Exploring Mediation of High School GPA*

INT = Preschool intervention. Est = Estimate. GPA = Grade point average. * *p <* .05

**Table S9.**

| Preschool | Gradeschool | High School | *Est.* | 95% CI |
| --- | --- | --- | --- | --- |
| Full 4-step Mediation Paths | | | | |
| INT -> Emotion Knowledge-> | Social Adjustment -> | Behavior Problems -> Graduation | .001 | .000, .003* |
| INT -> Emotion Knowledge-> | Parent Involvement -> | Behavior Problems -> Graduation | .000 | .000, .002* |
| INT -> Social Competence-> | Social Adjustment -> | Behavior Problems -> Graduation | .001 | .000, .005* |
| INT -> Social Competence-> | Parent Involvement -> | Behavior Problems -> Graduation | -.000 | -.001, .000 |
| INT -> Emotion Knowledge-> | Social Adjustment -> | Emotional Sympts-> Graduation | .000 | -.000, .001 |
| INT -> Emotion Knowledge-> | Parent Involvement -> | Emotional Sympts-> Graduation | .000 | -.000, .001 |
| INT -> Social Competence-> | Social Adjustment -> | Emotional Sympts-> Graduation | .000 | -.000, .001 |
| INT -> Social Competence-> | Parent Involvement -> | Emotional Sympts-> Graduation | -.000 | -.001, .000 |
| 3-step Mediation Paths (Without High School Behavior Problems) | | | | |
| INT -> Emotion Knowledge-> | Social Adjustment -> | Graduation | .002 | .000, .007* |
| INT -> Emotion Knowledge-> | Parent Involvement -> | Graduation | -.000 | -.002, .002 |
| INT -> Social Competence-> | Social Adjustment -> | Graduation | .004 | .000, .014* |
| INT -> Social Competence-> | Parent Involvement -> | Graduation | .000 | -.000, .002 |
| 3-step Mediation Paths (Without Preschool Effects) | | | | |
| INT -> | Social Adjustment -> | Behavior Problems -> Graduation | .001 | -.001, .008 |
| INT -> | Parent Involvement -> | Behavior Problems -> Graduation | .003 | .000, .010* |
| INT -> | Social Adjustment -> | Emotional Sympts-> Graduation | .000 | -.000, .002 |
| INT -> | Parent Involvement -> | Emotional Sympts-> Graduation | .001 | -.000, .007 |
| 2-step Mediation Paths (Without Behavior Problems or Preschool Effects) | | | | |
| INT -> | Social Adjustment -> | Graduation | .004 | -.003, .023 |
| INT -> | Parent Involvement -> | Graduation | -.001 | -.015, .009 |

*Serial Longitudinal Path Model Exploring Mediation of On-time Graduation*

INT = Preschool intervention. Est. = Estimate. Emotional Sympts= Emotional Symptoms. Graduation = on-time graduation. * *p <* .05

**Figure S1.**

Hypothesized Paths Linking Preschool Intervention to High School Outcomes: Saturated Model

| *Timeline: Preschool* | | *Gradeschool* | | | *High School*  *Grade 9 – 11 Cumulative* | | |
| --- | --- | --- | --- | --- | --- | --- | --- |
|  |  |  |  |  |  |  |  |
|  | Emotion Knowledge |  |  |  |  |  | GPA  On-time Graduation |
|  |  |  | Social Adjustment |  |  |  |  |
|  |  |  |  |  | Behavior Problems/ |  |  |
| REDI Intervention |  |  |  |  |  |  |  |
|  |  |  | Parent Involvement |  | Emotional Symptoms |  |  |
|  |  |  |  |  |  |  |  |
|  | Social- Competence |  | | |  |  |  |

Note: This figure shows the saturated model with all possible mediation paths tested. Subsequent figures show only the paths that were statistically significant. Model 1 tested mediation paths to high school behavior problems; model 2 tested mediation paths to high school emotional symptoms. Models 3 and 4 tested mediation paths to GPA and to On-time Graduation, included both high school behavior problems and emotional symptoms as potential mediators.
